# Vascular Endothelial Growth Factor Receptor Inhibitor SU5416 Suppresses Lymphocyte Generation and Immune Responses in Mice by Increasing Plasma Corticosterone

**DOI:** 10.1371/journal.pone.0075390

**Published:** 2013-09-16

**Authors:** Jamison J. Grailer, Douglas A. Steeber

**Affiliations:** Department of Biological Sciences, University of Wisconsin-Milwaukee, Milwaukee, Wisconsin, United States of America; UTHealth Medical School, United States of America

## Abstract

Inhibitors of vascular endothelial growth factor and its receptors (VEGFRs) are attractive therapeutic candidates for cancer treatment. One such small molecule VEGFR inhibitor, SU5416, limits angiogenesis in vivo and is widely used for investigating VEGFR signaling in tumor pathophysiology. Herein, we describe novel actions of SU5416 on the immune system. Treatment of mice with SU5416 for 3 days induced significant reductions in size and cellularity of peripheral lymph nodes. Interestingly, SU5416 did not affect initial lymphocyte localization to peripheral lymph nodes but did reduce lymphocyte accumulation during long-term migration assays. Treatment with SU5416 also induced severe loss of double-positive thymocytes resulting in thymic atrophy and a reduction in peripheral B cells. Furthermore, immune responses following immunization were reduced in mice treated with SU5416. Findings of thymic atrophy and reduced weight gain during SU5416 treatment suggested elevated corticosterone levels. Indeed, a significant 5-fold increase in serum corticosterone was found 4 hours after treatment with SU5416. Importantly, adrenalectomy negated the effects of SU5416 treatment on primary immune tissues, and partial reversal of SU5416-induced changes was observed following blockade of glucocorticoid receptors. SU5416 has been reported to inhibit the activation of latent transforming growth factor (TGF)-β, a cytokine involved in the regulation of glucocorticoid release by the adrenal glands. Interestingly, treatment with a TGF-β receptor inhibitor, showed a similar phenotype as SU5416 treatment, including elevated serum corticosterone levels and thymic atrophy. Therefore, these results suggest that SU5416 induces glucocorticoid release directly from the adrenal glands, possibly by inhibition of TGF-β activation.

## Introduction

Receptor tyrosine kinases (RTKs) are cell surface receptors that bind many polypeptides including hormones, cytokines, and growth factors. Upon activation by ligands, RTKs dimerize and autophosphorylate, initiating a downstream signaling cascade (reviewed in [1]). Inhibitors of RTKs are attractive therapeutics for cancer and other diseases due to their key role in the regulation of many cellular processes. However, due to the ubiquitous expression of RTKs, the potential for off-target effects is considerable. In this study, we describe significant off-target effects of a prominent RTK inhibitor, SU5416.

SU5416 (Semaxanib) was originally identified as a small-molecule inhibitor of vascular endothelial growth factor receptor (VEGFR)-2 [2]. Subsequently, it has been reported to inhibit several other RTKs including VEGFR-1, cKit, and Flt-3 [3], [4], [5]. However, SU5416 does exhibit considerable selectivity with respect to other RTKs, including epidermal growth factor receptor, insulin receptor, platelet-derived growth factor receptor-β, and fibroblast growth factor receptor [2]. SU5416 acts by reversibly blocking the ATP binding site of RTKs and inhibiting autophosphorylation, and does not affect VEGFR-2 surface expression or affinity for its ligand [6]. SU5416 has been demonstrated to be anti-angiogenic in vivo [7], and treatment with SU5416 decreased the size and vascularity of tumors in many murine cancer models [2]. Despite promising results in preclinical trials as an anti-cancer therapeutic, SU5416 has demonstrated limited success in clinical trials [8], [9], [10]. In fact, phase III trials of SU5416 in patients with advanced colorectal cancer were cut short due to limited clinical benefit [11]. Despite cessation as a potential drug candidate, SU5416 remains widely used as an investigative tool for the study of RTKs, and in particular, VEGFR signaling and function.

Interestingly, SU5416 has been reported to inhibit the function of tissue transglutaminase, an enzyme important for the conversion of transforming growth factor (TGF)-β from a latent to a bioactive form [12]. Importantly, TGF-β1 regulates the release of corticosterone from the adrenal glands (reviewed in [13]). Therefore, alterations in TGF-β activation has the potential to influence corticosterone release from the adrenal glands. Since corticosterone is a potent anti-inflammatory mediator (reviewed in [14]), enhanced release of corticosterone can significantly alter immune responses in humans and animal models.

Previously, we utilized SU5416 during studies of angiogenesis in lymphoid tissues (JJG and DAS, manuscript in preparation) and noted potential immune side effects. Furthermore, anomalies in leukocyte homeostasis, including lymphopenia, have been observed during clinical trials of SU5416 [15], [16], [17]. However, the effects of SU5416 on the immune system have not been studied. Therefore, the present study investigated effects of SU5416 treatment on immune system homeostasis and immune responses in mice. The results of these studies suggest that treatment with SU5416 generates increased serum corticosterone levels, decreased lymphocyte production and reduced immune responses. Although we cannot confirm a mechanism, we provide evidence that SU5416 induces blockade of TGF-β activation in the adrenals, which leads to increased corticosterone release.

## Materials and Methods

### Animals

C57BL/6 mice were purchased from The Jackson Laboratory (Bar Harbor, ME). Surgically adrenalectomized mice (C57BL/6) were purchased from Charles River Laboratories (Wilmington, MA). Adrenalectomized mice were maintained on isotonic saline and used within 10 days of arrival. All mice used were 2–4 months of age and were housed in a specific pathogen-free barrier facility with unrestricted access to food and water. All studies and procedures were in accordance with NIH guidelines and were approved by the Animal Care and Use Committee of the University of Wisconsin-Milwaukee under protocol 12–13 #07.

### VEGFR, Glucocorticoid Receptor, TGF-β Receptor, and VEGF Inhibitor Treatments

The VEGFR inhibitor SU5416 (Z-3-((2,4-dimethylpyrrol-5-yl)methylidenyl)-2-indolinone), the TGF-β receptor inhibitor SB431542, and the glucocorticoid receptor inhibitor RU486 (all from Sigma, St. Louis, MO) were dissolved in dimethyl sulfoxide (DMSO) at a stock concentration of 20 mg/mL and stored at –20°C until use. SU5416 was administered intraperitoneally (i.p.) at the indicated doses and times listed for each experiment. SB431542 was administered i.p. at a dose of 25 mg/kg/day. RU486 was administered i.p. at a dose of 50 mg/kg/day. An equivalent dose of DMSO was used as vehicle control for all three inhibitors. Bevacizumab (trade name Avastin®; Roche, Basel, Switzerland), a humanized monoclonal antibody specific to VEGF-A, is a prominent FDA-approved VEGF-directed therapy used for the treatment of several cancer types and was administered i.p. at 250 µg/mouse. This dose has previously been reported to be biologically active in mouse models of tuberous sclerosis and acute colitis, and was reported to be a direct inhibitor of murine angiogenesis and lymphangiogenesis [18], [19], [20]. Purified human IgG (Hu IgG, 250 µg/mouse; Jackson Immunoresearch, West Grove, PA) was used as an irrelevant control antibody.

### Immunization Studies

Mice were immunized subcutaneously unilaterally in the fore- and hind-limb with Imject® Alum- (Pierce, Rockford, IL) precipitated keyhole limpet hemocyanin (KLH-Alum, 200 µg KLH/injection site; EMD, Gibbstown, NJ). The opposite side limbs were used as an internal contra-lateral control. For all studies, the control or draining axillary and popliteal peripheral lymph nodes (PLN) were analyzed. Mice were treated with 25 mg/kg/day SU5416 starting on the day of immunization. Three days following immunization, control and draining PLN were harvested and weighed on a digital scale (Mettler-Toledo AB54-S; Columbus, OH). Single-cell suspensions were prepared as previously described [21] and cell numbers were enumerated using a hemocytometer.

### In vivo Migration Assays

Mice were immunized with KLH-Alum as described above. For short-term (1 hour) migration assays, mice were treated with 25 mg/kg/day SU5416 or vehicle starting on the day of immunization. In separate short-term migration experiments, mice were treated with 1 dose of bevacizumab or Hu IgG on the day of immunization. For long-term (48 hour) migration assays, mice were treated with 50 mg/kg/day SU5416 or vehicle on the day of immunization and 3 days following immunization. Importantly, twice-weekly SU5416 doses of 50 mg/kg have been reported to have the same efficacy as a daily dose of 25 mg/kg [6]. In separate long-term migration experiments, mice were treated with a single dose of bevacizumab or Hu IgG on the day of immunization and again 3 days following immunization. Three days following immunization, splenocytes from donor mice were isolated and erythrocytes in the preparations were lysed with a 0.1 M ammonium chloride solution. The cells were then labeled for tracking and 30–40×10^6^ cells in 300–400 µl were injected via the lateral tail vein into immunized, drug- or vehicle-treated recipient mice. For short-term assays, splenocytes were labeled with EZ link-sulfo-NHS-biotin (80 µg/mL; Pierce) for 15 min at room temperature. For long-term migration experiments, cells were labeled with carboxyfluorescein diacetate succinimydl ester (CFSE, 0.2 µM; Molecular Probes, Eugene, OR) for 30 minutes at 37°C followed by a 30 minute incubation at 37°C in RPMI containing 10% FBS to ensure complete modification of the label. CFSE was used so that cell proliferation could also be tracked as previously described [22]. One or 48 hours after cell transfer, single-cell suspensions were prepared from the control and draining PLN of recipient mice. Preinject and recipient single-cell suspensions were labeled with fluorochrome-conjugated antibodies to detect CD4, CD8, CD19 (all from BD Biosciences, San Jose, CA), and CD44 (eBioscience, San Diego, CA). Cell preparations from short-term assays were also labeled with fluorochrome-conjugated avidin (Invitrogen, Carlsbad, CA) to detect biotinylated cells. Irrelevant isotype-matched fluorochrome-conjugated antibodies were used to determine background staining (Southern Biotech, Birmingham, AL). Cells were analyzed on a FACSCalibur flow cytometer (BD Biosciences) by gating on cells having the forward and side light scatter properties of lymphocytes. The total number of transferred cells recovered from individual lymphoid tissues was determined by multiplying the total cell counts for individual tissues by the frequency of transferred cells. The percentage of the injected population found in each tissue (percent of injected) was determined by dividing the number of transferred cells in each tissue by the number that were injected and multiplying by 100.

### Lymphocyte Subset Analysis

Mice were treated with 25 mg/kg/day SU5416 or vehicle control for 3 days. In separate experiments, mice were treated with a single dose of bevacizumab or Hu IgG and tissues were harvested 3 days later. Spleen, PLN (1 axillary and 1 popliteal lymph node were pooled), thymus, and bone marrow were collected, single cells prepared, enumerated and the frequency of cell subpopulations was determined by flow cytometry as above. For analysis, cells were labeled with tissue-appropriate fluorochrome-conjugated antibodies to detect CD4, CD8, CD19, CD44, B220, and IgM, and biotinylated anti-mouse IgD (all from BD Biosciences) followed by fluorochrome-conjugated avidin (eBioscience). The total number of each subpopulation was determined by multiplying the total cell count by the frequency of each subpopulation found in the tissue. In some experiments, mice were treated with RU486 at the time of SU5416 treatment.

In a separate set of experiments, thymic cell numbers were determined as described above after 3 days of treatment with SB431542 (25 mg/kg/day) or vehicle control.

### Mouse Weight Measurements

Mice were treated with 50 mg/kg SU5416 or vehicle control twice per week for 3 weeks. Mice were weighed on a digital scale and the percent change in weight was calculated for individual mice and averaged.

### Corticosterone ELISA

Mice were treated with one dose of 25 mg/kg SU5416 or vehicle control and bled via the retro-orbital venous plexus 4 or 24 hours later. In separate experiments, mice were treated with one dose of 25 mg/kg SB431542 or vehicle control with blood being collected 1.5 or 4 hours later. Sex-matched mice from all groups were housed identically (same-sex housing, 12 h light/dark cycle) in a dedicated animal facility free from uncontrolled human traffic. Mice were anesthetized briefly by inhalation of isoflurane and bled via the retro-orbital venous plexus. Serum was collected from separate mice for each time point, and the drug treatments were staggered so that serum was collected at the same time of day for all mice. Serum was analyzed using a competitive ELISA kit (Caymen Chemical, Ann Arbor, MI) to detect corticosterone.

### Analysis of Cellular Immune Response

Mice were immunized with KLH-Alum as described above, and were treated with 25 mg/kg/day SU5416 or vehicle control starting on the day of immunization. Three days following immunization, the mice were pulsed for 1 hour with 5-bromo-2-deoxyuridine (BrdU, 100 µg/g body weight, administered i.p.; Sigma). Control and draining PLN were harvested, and single-cell suspensions were labeled with fluorochrome-conjugated anti-mouse CD45 antibody (pan leukocyte marker, eBioscience), and treated with ice-cold 70% EtOH for 30 minutes at 4°C followed by incubation in 1% formaldehyde with 0.1% Tween 20 for 30 minutes at room temperature. Cells were then treated with 50 KU/mL DNase I (Sigma) for 10 minutes at room temperature, labeled with fluorochrome-conjugated anti-BrdU antibody (BD Biosciences), and analyzed by flow cytometry. The number of BrdU^+^ cells was determined by multiplying the frequency found in the tissue by the total cell count.

### Analysis of Humoral Immune Response

Mice were immunized in all limbs with Imject® Alum-precipitated dinitrophenyl-KLH (DNP-KLH-Alum, 25 µg KLH per injection site; Sigma). Mice were treated with 50 mg/kg SU5416 or vehicle control twice weekly for 3 weeks starting on the day of immunization. Serum was collected 0, 7, 14, and 21 days following immunization. Mice were not treated with SU5416 or vehicle for 1 week starting on day 21. Mice were boosted with DNP-KLH-Alum on day 28, and SU5416 or vehicle control treatment was resumed for 1 week on the day of the boost. Serum was again collected on day 35 (1 week after boost). DNP-specific IgM and IgG1 serum antibody levels were determined by isotype-specific ELISA as described [23] using 96-well plates coated with DNP-conjugated bovine serum albumin (CalBiochem/EMD, San Diego, CA).

### Statistical Analysis

Results are shown as mean ± SEM. The data were analyzed using nonparametric tests after discovering ANOVA assumptions (normality of the residuals and homogeneity of variances) failed in many cases. Significant differences between sample means were determined using individual Kruskal-Wallis tests (SAS Institute Inc., Cary, NC). A p value <0.05 was considered to be significant.

## Results

### SU5416 Reduces Resting and Activated PLN Weight and Cellularity

**Figure 1:**
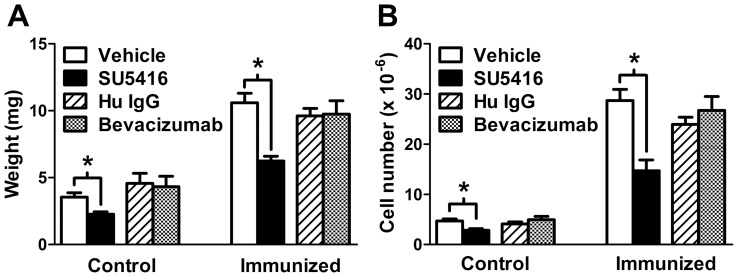
Treatment with SU5416 reduces PLN weight and cellularity. Mice were immunized unilaterally in the fore- and hind-limb with KLH-Alum and treated with SU5416 (25 mg/kg/day) or vehicle for 3 days starting on the day of immunization. In separate experiments, mice were immunized and treated with bevacizumab or Hu IgG control, and PLN were harvested on day 3. Bar graphs show the mean ± SEM values for A) wet weight and B) total cell number from control (contra-lateral) and draining PLN from 6–10 mice per group. *Differences in the mean values between SU5416 and vehicle control treatments were significant; p<0.05.

Subcutaneous immunization with KLH-Alum resulted in 3.1- and 5.6-fold increases in PLN weight and cellularity, respectively (Fig. 1, vehicle control bars). Importantly, VEGF has been reported to play a key role in the regulation of PLN vasculature during an immune response [24]. Consistent with this, even though PLN from day 3 KLH-Alum immunized mice treated with the VEGFR inhibitor SU5416 were larger than their unimmunized counterparts, they were noticeably smaller in size than immunized PLN from vehicle-treated mice, which correlated with reductions in both the weight (by 41%) and cellularity (by 45%) of the tissues (Fig. 1). Interestingly, PLN from non-immunized SU5416-treated mice showed similar reductions in weight (34%) and cellularity (41%). To confirm these effects were VEGF dependent, control and immunized mice were treated with the VEGF-blocking antibody bevacizumab or Hu IgG control. Surprisingly, treatment with bevacizumab had no effect on PLN weight or cellularity from either control or immunized mice (Fig. 1). Therefore, these results suggest that the observed effects of SU5416 treatment on the PLN did not result from blockade of VEGF-mediated signaling.

### SU5416 Treatment Reduces Lymphocyte Accumulation in PLN

**Figure 2:**
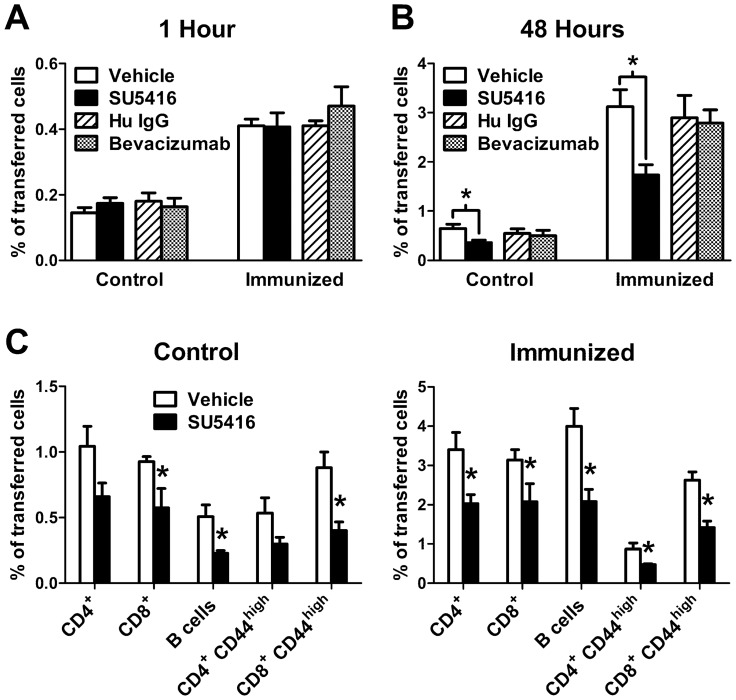
SU5416 treatment reduces lymphocyte accumulation in PLN. Recipient mice were immunized as described in Fig. 1. A) Mice were treated with SU5416 (25 mg/kg/day) or vehicle, or bevacizumab or Hu IgG starting on the day of immunization. Three days following immunization, donor splenocytes were biotinylated and transferred into the above treated recipient mice. One hour after transfer, control and draining PLN were harvested, and single-cell suspensions were labeled with fluorochrome-conjugated avidin to detect transferred biotinylated cells and analyzed by flow cytometry. B) Mice were treated as above but the migration of CFSE-labeled lymphocytes was analyzed 48 hours following transfer into recipient mice. C) Experiments (48 hour migration assays) were performed as described in B and further analyzed to determine lymphocyte subsets. Specifically, recipient cell suspensions were labeled with fluorochrome-conjugated antibodies to detect CD4, CD8, CD19 (B cells), and CD44 (CD44^high^ effector/memory cells), and analyzed by flow cytometry. Values indicate the mean ± SEM percent of adoptively transferred cells (% of transferred cells) that were recovered from each tissue from 3–4 mice per group. *Differences in the mean values between SU5416 and vehicle control treatments were significant; p<0.05.

The above results demonstrated that treatment with SU5416 for 3 days caused reduced PLN cellularity. A major factor influencing PLN cellularity is lymphocyte migration into the tissue, which occurs during the process of lymphocyte recirculation and increases significantly during an immune response [25], [26]. Therefore, the effects of SU5416 treatment on lymphocyte recruitment into resting and immunized PLN were examined. Splenocytes were labeled with biotin (1 hour assays) or CFSE (48 hour assays) and adoptively transferred into SU5416- or vehicle-treated recipient mice. In separate experiments, mice were treated with bevacizumab or Hu IgG prior to adoptive cell transfer. Treatment of recipient mice with SU5416 or bevacizumab did not reduce lymphocyte migration into resting or immunized PLN during short-term assays (Fig. 2A). However, during long-term assays, treatment with SU5416 reduced lymphocyte accumulation in resting and immunized PLN by 44% and 46%, respectively (Fig. 2B). Furthermore, SU5416 treatment reduced the accumulation of virtually all lymphocyte subsets into control and immunized PLN by 34–55%, including effector/memory phenotype (CD44^high^) populations of CD4^+^ and CD8^+^ T cells (Fig. 2C). Importantly, only negligible levels (<0.1%) of cell proliferation were observed in the migrated cells from either SU5416- or vehicle-treated mice during long-term assays, as assessed by CFSE fluorescence intensity measurements (data not shown). By contrast, blockade of VEGF with bevacizumab had no effect on lymphocyte migration during long-term assays (Fig. 2B). Therefore, treatment with SU5416 reduced lymphocyte accumulation in the PLN independent of VEGF function.

### Treatment with SU5416 Induces Loss of Lymphocytes in Primary and Secondary Lymphoid Tissues

**Figure 3:**
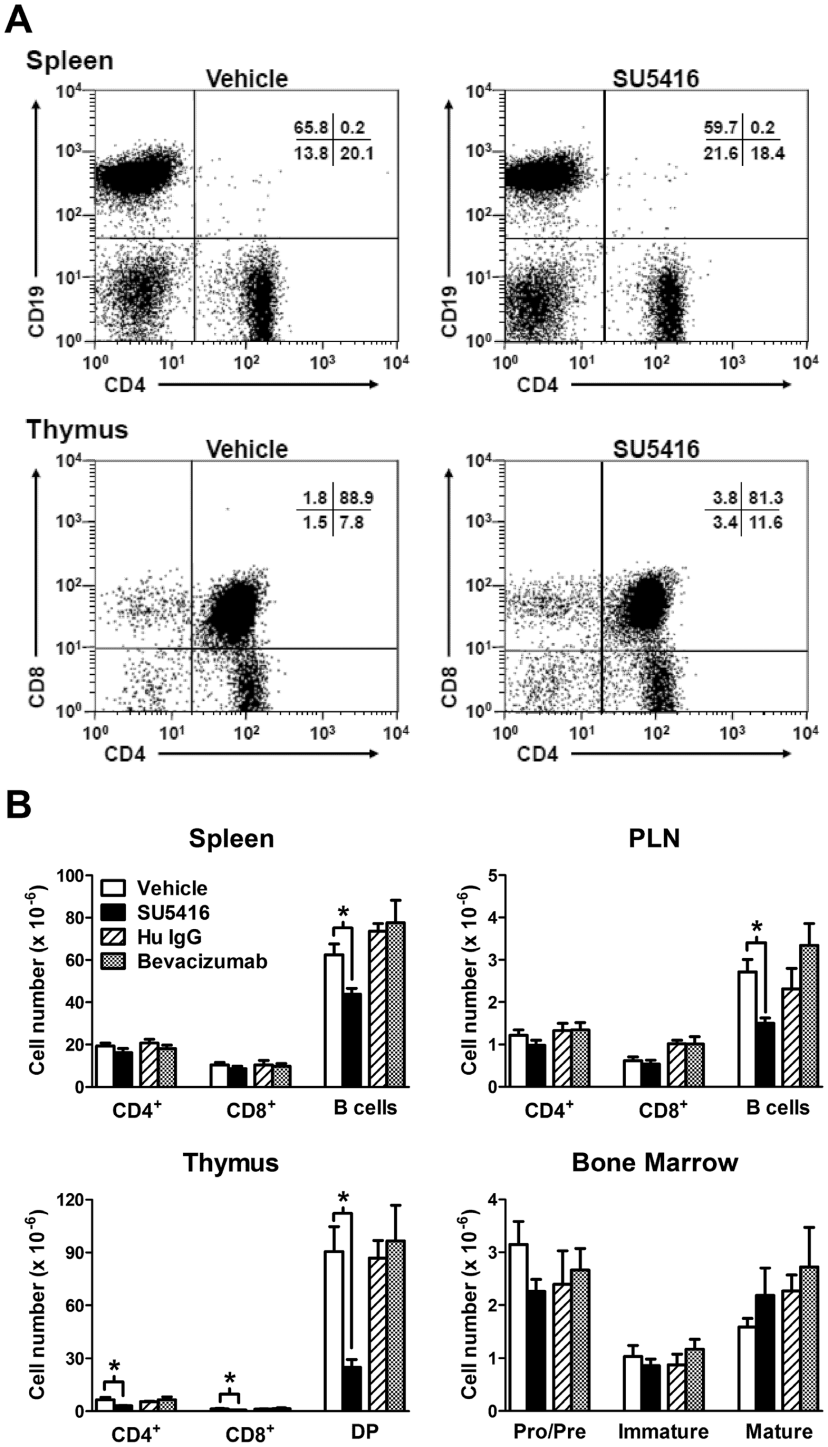
Loss of lymphocytes from lymphoid tissues following SU5416 treatment. Mice were treated with SU5416 (25 mg/kg/day) or equivalent amounts of vehicle. In separate experiments, mice were treated with bevacizumab or Hu IgG. Tissues were harvested 3 days later and labeled for flow cytometric analysis. A) Representative flow cytometry dot plots of spleen and thymus from SU5416 and vehicle control treated mice. Numbers indicate the frequency of cells located in the corresponding quadrants. B) Values represent the mean ± SEM number of cells from 4–9 mice per tissue and group. DP, CD4 and CD8 double-positive; Pro/Pre, progenitor/precursor B cells. *Differences in the mean values between SU5416 and vehicle control treatments were significant; p<0.05.

Since treatment with SU5416 reduced the accumulation of all lymphocyte subsets in the PLN during in vivo migration experiments, effects of SU5416 treatment on lymphocyte populations in the secondary lymphoid tissues (PLN and spleen) under homeostatic (non-immunized) conditions were examined. Results showed that three days of treatment with SU5416 reduced total spleen cellularity by 25% (Table 1). Interestingly, SU5416 had little effect on the number or frequency of T cell subsets found in the spleen (Fig. 3 and Table 1). In contrast, B cell numbers were significantly reduced (by 30%) in the spleen compared to vehicle-treated controls (Fig. 3B). Within the PLN, the frequency of B cells was significantly decreased compared to vehicle-treated controls, which corresponded to a 45% reduction in the number of total B cells (Table 1, Fig. 3B). The reduced frequency of B cells in the SU5416-treated PLN resulted in a concomitant significant increase in CD4^+^ T cell frequency (Table 1). The number of CD44^high^ effector/memory phenotype T cells in the spleen or PLN was not affected by SU5416 treatment (data not shown). Therefore, treatment with SU5416 induced a rapid depletion of peripheral B cells. By contrast, bevacizumab treatment had no effect on peripheral B cells (Fig. 3B, Table 1).

**Table 1:** Lymphocyte subset tissue cell counts and frequencies^a^.

	Cell Number (× 10^–6^)	Frequency (%)		
Spleen		CD4^+^	CD8^+^	B cells
Vehicle	96.3±5.2	20±2	11±1	64±3
SU5416	72.2±4.1*	22±2	12±1	61±3
Hu IgG^b^	108±4.2	20±1	10±2	68±3
Bevacizumab	109±9.8	17±3	9±2	71±4
**PLN**		**CD4^+^**	**CD8^+^**	**B cells**
Vehicle	4.7±0.4	26±1	14±2	58±3
SU5416	3.0±0.3*	32±2*	18±2	50±3*
Hu IgG	4.7±0.7	29±3	22±2	48±5
Bevacizumab	5.8±0.8	24±1	18±2	58±3
**Thymus**		**CD4^+^**	**CD8^+^**	**DP**
Vehicle	101±16	6.6±1	1.3±0.2	90±1
SU5416	29.9±4.5*	11±2*	2.4±0.5*	82±3*
Hu IgG	96.7±10	5.8±1	1.3±0.2	90±1
Bevacizumab	109±23	6.1±1	1.3±0.2	89±1
**BM** c		**Pro/Pre**	**Imm**	**Mature**
Vehicle	6.8±0.8	46±2	15±1	24±3
SU5416	7.1±1.1	33±3*	12±1	31±4
Hu IgG	6.8±0.9	35±3	13±1	34±2
Bevacizumab	7.9±1.0	34±1	15±1	34±3

a Mice were treated with SU5416 (25 mg/kg/day) or equivalent amounts of vehicle control for 3 days. In separate experiments, mice were treated with bevacizumab or Hu IgG. Tissues were harvested after 3 days and labeled for flow cytometric analysis. Spleen and PLN were labeled for detection of CD4, CD8, and CD19 (B cells). Thymus was labeled for CD4 and CD8. Bone marrow was labeled for B220, IgM, and IgD. *Differences between vehicle and SU5416 were significant; p<0.05.

b Abbreviations used: Hu IgG, human IgG control; PLN, peripheral lymph nodes; DP, CD4 and CD8 double-positive; BM, bone marrow; Pro/Pre, IgM^-^IgD^–^ progenitor/precursor B cells; Imm, IgM^+^IgD^–^ immature B cells; Mature, IgM^+^IgD^+^ mature B cells.

c Frequencies and total number of B cell populations in BM were calculated by gating on B220^+^ cells.

To determine whether any of the above results were due to effects of SU5416 treatment on lymphocyte development, cell populations within the primary lymphoid tissues, thymus and bone marrow, were examined. Strikingly, 3 days of treatment with SU5416 induced a severe loss of thymocytes (Table 1). The number of CD4^+^ CD8^+^ double-positive (DP) lymphocytes in the thymus was reduced (by >70%) compared to vehicle-treated controls (Fig. 3B). SU5416 treatment also significantly reduced the number of CD4^+^ and CD8^+^ single-positive (SP) T cells by 56% and 53%, respectively (Fig. 3B). When subset frequencies were analyzed, the frequency of DP thymocytes was significantly reduced while the frequencies of the SP T cells were increased (Table 1 and Fig. 3A). This result suggests that SU5416 treatment had a greater relative effect on the DP thymocytes. Interestingly, the number of DP thymocytes in SU5416-treated mice was already reduced by 54% compared to vehicle-treated controls following only 2 days of SU5416 treatment (p<0.05, data not shown). These results indicate that SU5416 treatment induces rapid and dramatic loss of thymocytes. In contrast to SU5416, treatment with bevacizumab did not affect the number or composition of thymocytes compared to Hu IgG–treated controls (Fig. 3B and Table 1).

In contrast to the thymus, SU5416 had more modest effects on cell populations in the bone marrow. SU5416 did not affect the number of B220^+^ cells in the bone marrow (Table 1). The most notable effect on the bone marrow following 3 days of SU5416 treatment was a significant 28% reduction in the frequency of progenitor/precursor (pro/pre) B cells compared to vehicle-treated controls (Table 1). This decreased frequency of pro/pre B cells correlated with a 28% decrease in the total number of these cells although this did not reach statistical significance (p = 0.09, Fig. 3B). In addition, the frequencies of immature and mature B cells trended toward a 20% decrease and 29% increase, respectively, compared to vehicle-treated controls (Table 1, p = 0.07 and p = 0.06, respectively). As before, treatment with bevacizumab did not result in any changes in bone marrow B cell number or composition (Fig. 3B and Table 1), indicating that blockade of VEGF does not negatively impact B cell development. Notably, SU5416 had no effect on the number or frequency of Gr1^+^, F4/80^+^, or CD11b^+^ granulocytes/monocytes in the bone marrow (data not shown). Taken together, these results indicate that SU5416 treatment negatively affects both B and T cell development.

### Treatment with SU5416 Inhibits Normal Weight Gain

**Figure 4:**
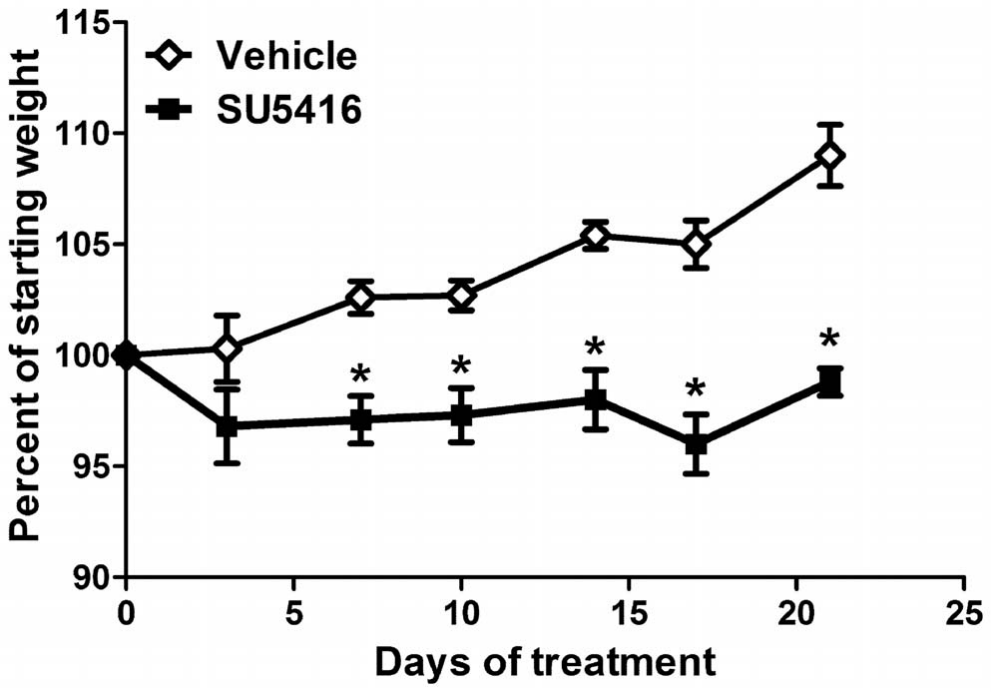
SU5416 treatment inhibits weight gain. Mice were treated with 50/kg SU5416 or vehicle twice per week for 3 weeks. Mice were weighed twice per week. Values represent the mean ± SEM percentage of weight change from 5 mice per group. *Differences in the mean values between SU5416 and vehicle control treatments were significant; p<0.05.

The effects of SU5416 treatment on age-dependent weight gain in mice were assessed over a 3-week period. Specifically, mice were treated with 50 mg/kg SU5416 or vehicle control twice a week for three weeks. In general, vehicle-treated animals continued to gain weight throughout the time course, with weight increasing 9% over starting weight by day 21 (Fig. 4). In contrast, SU5416-treated animals failed to gain weight, and in fact, lost weight rapidly, reaching a maximum 4% decrease in weight by day 17. Therefore, treatment with SU5416 significantly affected normal weight gain in mice.

### SU5416 Triggers Corticosterone Release from the Adrenal Glands

**Figure 5:**
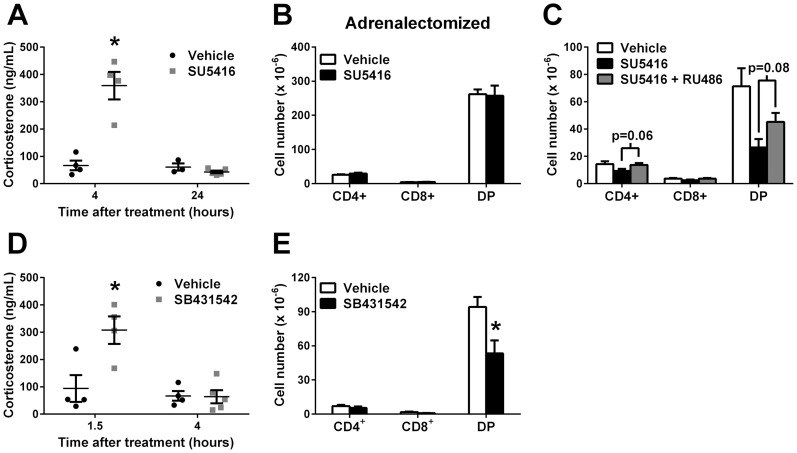
SU5416 treatment transiently elevates serum corticosterone through inhibition of TGF-β activation. A) Mice were treated with 25 mg/kg SU5416 or vehicle control, serum was collected 4 or 24 hours later and the level of corticosterone was measured by competitive ELISA. B) Adrenalectomized mice were treated with 25 mg/kg/day SU5416 or vehicle control for 3 days. Thymocyte subsets were determined following labeling with fluorochrome-conjugated antibodies to detect CD4 and CD8 and flow cytometry analysis. Values indicate the mean ± SEM cell number of each subset from 5–6 mice per group. C) Mice were treated with 25 mg/kg/day SU5416 or vehicle in the absence or presence of 50 mg/kg/day RU486 for 3 days. Thymocyte subsets were determined as described in B. Values indicate the mean ± SEM cell number of each subset from 5–6 mice per group. D) Mice were treated with 25 mg/kg SB431542 or vehicle control for 1.5 or 4 hours and the level of corticosterone was measured as above. For both A and C, symbols represent results from individual animals and the horizontal lines indicate the mean ± SEM concentration of corticosterone in each group from 4–5 mice per group. E) Mice were treated with 25 mg/kg/day SB431542 or vehicle control for 3 days. Thymocyte subsets were determined as above. Values indicate the mean ± SEM cell number of each subset from 5–7 mice per group. *Differences in the mean values between SU5416 or SB431542 and vehicle control treatments were significant; p<0.05.

The above findings of decreased lymphocyte production and reduced weight gain during SU5416 treatment suggested elevated corticosterone levels [27], [28]. Therefore, sex-matched mice were treated with SU5416 or vehicle control and serum corticosterone levels were analyzed as detailed in the Materials and Methods section. Indeed, SU5416 treatment induced a rapid and transient increase in serum corticosterone. Specifically, 4 hours following SU5416 treatment corticosterone levels were increased by 5.4-fold relative to vehicle-treated controls but returned to basal levels by 24 hours (Fig. 5A). In addition, equivalent increased levels of serum corticosterone were found as long as 8 hours following SU5416 treatment (SU5416, mean ± SD, 360±140 ng/mL; n = 2). Therefore, a dramatic but transient increase in serum corticosterone levels occurred following treatment with SU5416.

Surgically adrenalectomized mice were utilized to determine if SU5416 affected immune tissues via the adrenal glands. Adrenalectomized mice were treated with SU5416 (25 mg/kg/day) for 3 days and the effects on primary and secondary immune tissues were determined. Importantly, adrenalectomy induced thymic hyperplasia, as previously reported (compare Table 1 and Table 2, p<0.05, Ref. [29]). Results showed that adrenalectomized mice displayed a lack of response in the thymus to SU5416. Specifically, the number and frequency of thymocyte subsets were unchanged by treatment with SU5416 (Fig. 5B and Table 2). Furthermore, there was no observable effect on bone marrow subset composition or cell number (Table 2 and data not shown).

**Table 2:** Tissue cell counts and frequencies in adrenalectomized mice^a^.

	Cell Number (× 10^−6^)	Frequency (%)		
Thymus		CD4^+^	CD8^+^	DP^b^
Vehicle	294±16	8.7±0.1	1.6±0.1	89.1±1
SU5416	293±32	11±0.9	1.8±0.3	87.0±1
**BM** c		**Pro/Pre**	**Imm**	**Mature**
Vehicle	8.9±0.7	64±3	19±1	16±2
SU5416	7.7±1.2	64±4	17±2	18±3
**Spleen**		**CD4^+^**	**CD8^+^**	**B cells**
Vehicle	180±18	25±2	15±1	53±3
SU5416	132±11*	27±2	15±2	52±3
**PLN**		**CD4^+^**	**CD8^+^**	**B cells**
Vehicle	6.4±0.6	46±3	40±2	12±2
SU5416	8.1±0.8	46±1	36±2	17±2

a Surgically adrenalectomized mice were treated with SU5416 (25 mg/kg/day) or equivalent amounts of vehicle. Tissues were harvested after 3 days and labeled for flow cytometric analysis. Spleen and PLN were labeled for detection of CD4, CD8, and CD19 (B cells). Thymus was labeled for CD4 and CD8. Bone marrow was labeled for B220, IgM, and IgD. *Differences between vehicle and SU5416 were significant; p<0.05.

b Abbreviations used: DP, CD4 and CD8 double-positive; BM, bone marrow; PLN, peripheral lymph node; Pro/Pre, IgM^-^IgD^–^ progenitor/precursor B cells; Imm, IgM^+^IgD^–^ immature B cells; Mature, IgM^+^IgD^+^ mature B cells.

c Frequencies and total cell number of B cell populations in BM were calculated by gating on B220^+^ cells.

SU5416 did have a modest effect on the spleen in adrenalectomized animals. Specifically, total splenocyte number was reduced by 26% (Table 2). There was a significant decrease in the number of CD8^+^ T cells (by 25%) although reductions in other subsets nearly achieved statistical significance (p = 0.08 and p = 0.10 for CD4^+^ T cells and B cells, respectively, data not shown). It is important to note that this CD8-specific effect was not observed in adrenal-sufficient mice following SU5416 treatment, and could be the result of an independent mechanism that was masked by the glucocorticoid-dependent effects. However, SU5416 treatment had no effect on total numbers of lymphocyte subsets in the PLN of adrenalectomized mice (Table 2). Taken together, these results indicate that SU5416 induces glucocorticoid release from the adrenal glands, which affects lymphocyte populations in both primary and secondary lymphoid tissues.

In order to further confirm a role for glucocorticoids in the effects of SU5416 treatment, the glucocorticoid receptor inhibitor RU486 [30] was used. Specifically, mice were dosed with SU5416 (25 mg/kg/day) in the presence or absence of RU486 (50 mg/kg/day) for three days. Primary and secondary tissues were analyzed as described above. In the thymus, blockade of glucocorticoid receptors resulted in a modest reversal of the effects of SU5416 on double positive thymocytes, although this did not reach statistical significance (Fig. 5C). However, glucocorticoid receptor blockade fully reversed the effects of SU5416 on peripheral tissues (Table 3) resulting in significant differences in cell numbers between SU5416- and SU5416+ RU486-treated mice (spleen, p<0.005; PLN, p<0.05). These results indicate that the effects of SU5416 are at least partially dependent on glucocorticoid receptors.

**Table 3:** Tissue cell counts and frequencies in SU5416 and RU486 treated mice^a^.

	Cell Number (× 10^–6^)	Frequency (%)		
Thymus		CD4^+^	CD8^+^	DP^b^
Vehicle	91±15	16±2	4.3±0.8	78±3
SU5416	39±8*	25±4	6.2±0.7	66±4*
SU+RU486	64±7	22±1	5.9±0.9	70±2
**BM** c		**Pro/Pre**	**Imm**	**Mature**
Vehicle	5.2±0.4	55±5	12±1	32±6
SU5416	5.4±0.6	44±2	10±1	45±3
SU+RU486	3.2±0.5*	69±3*	15±1	16±2*
**Spleen**		**CD4^+^**	**CD8^+^**	**B cells**
Vehicle	95±4	29±1	19±1	43±2
SU5416	62±6*	32±1*	19±1	40±1
SU+RU486	98±6	29±1	17±1	47±1*
**PLN**		**CD4^+^**	**CD8^+^**	**B cells**
Vehicle	1.8±0.2	45±3	40±1	13±3
SU5416	1.2±0.2^†^	46±1	42±2	10±2
SU+RU486	2.0±0.3	45±2	43±1	11±1

a Mice were treated with SU5416 (25 mg/kg/day) or equivalent amounts of vehicle. Some SU5416-treated mice were also treated with RU486 (50 mg/kg/day). Tissues were harvested after 3 days and labeled for flow cytometric analysis. Spleen and PLN were labeled for detection of CD4, CD8, and CD19 (B cells). Thymus was labeled for CD4 and CD8. Bone marrow was labeled for B220, IgM, and IgD. *Differences between vehicle and inhibitor-treated tissues were significant; p<0.05. ^†^p = 0.052 vs. vehicle control.

b Abbreviations used: DP, CD4 and CD8 double-positive; BM, bone marrow; PLN, peripheral lymph node; Pro/Pre, IgM^-^IgD^–^ progenitor/precursor B cells; Imm, IgM^+^IgD^–^ immature B cells; Mature, IgM^+^IgD^+^ mature B cells.

c Frequencies and total cell number of B cell populations in BM were calculated by gating on B220^+^ cells.

### Treatment with a TGF-β Receptor Inhibitor Induces an Acute Increase in Serum Corticosterone and Loss of DP Thymocytes

SU5416 is a promiscuous RTK inhibitor and has been reported to block tissue transglutaminase in vitro, an enzyme important for the conversion of TGF-β from a latent to a bioactive form [12]. Of note to the present studies, TGF-β1 is an important cytokine regulating the release of corticosterone from the adrenal glands (reviewed in [13]). Therefore, it is possible that the increased serum corticosterone levels were due to blockade of TGF-β1 activation in the adrenal glands. An attempt was made to detect latent and active TGF-β in mouse adrenal glands using a commercially available ELISA kit (R & D Systems) following SU5416 treatment. Unfortunately, despite pooling adrenal homogenates from multiple animals, detectable levels of active TGF-β were not obtained even from control treated animals.

As an alternative approach to directly measuring levels of latent and active TGF-β, direct blockade of TGF-β receptors with the small-molecule inhibitor SB431542 [31] was used. Specifically, mice were treated with 25 mg/kg SB431542 or vehicle control and serum was collected 1.5 and 4 hours later. Following 1.5 hours of SB431542 treatment, a 3.3-fold increase in corticosterone levels relative to vehicle-treated controls was observed (Fig. 5D). However, corticosterone levels were already reduced to basal levels by 4 hours after SB431542 treatment. Therefore, SB431542 treatment induced a rapid and transient increase in serum corticosterone levels.

To determine whether the increased serum corticosterone levels observed following SB431542 treatment impacted lymphoid tissues, lymphocyte subset composition was examined. Mice were treated with 25 mg/kg/day SB431542 or vehicle control for 3 days following which primary and secondary lymphoid tissues were analyzed by flow cytometry as described above. Similar to SU5416, treatment with SB431542 reduced the number of DP thymocytes by 43% compared to vehicle-treated controls (Fig. 5E). Furthermore, SB431542 treatment reduced the frequency of DP thymocytes by a modest, but significant, 4% (p<0.05, data not shown), and induced a concomitant increase in the frequency of CD4^+^ SP T cells by 30% (p<0.05, data not shown). Interestingly, while no significant changes in the frequency or number of lymphocyte subsets in the spleen or PLN were observed, SB431542 treatment did tend to reduce both the frequency (by 9%) and number (by 19%) of B cells in the PLN (data not shown). However, no significant changes in bone marrow B cell subsets were found following treatment with SB431542. Therefore, treatment with SU5416 or SB431542 resulted in transient elevation of serum corticosterone and loss of DP thymocytes. The more modest effects of SB431542 treatment on lymphoid tissue cellularity than those of SU5416 treatment may be a result of a smaller window of elevated corticosterone levels (compare Figs. 5A and 5D). Taken together, these results suggest that SU5416 may mediate its effects through inhibition of TGF-β activation in the adrenal glands.

### SU5416 Treatment Reduces Immunization-induced Immune Response

**Figure 6:**
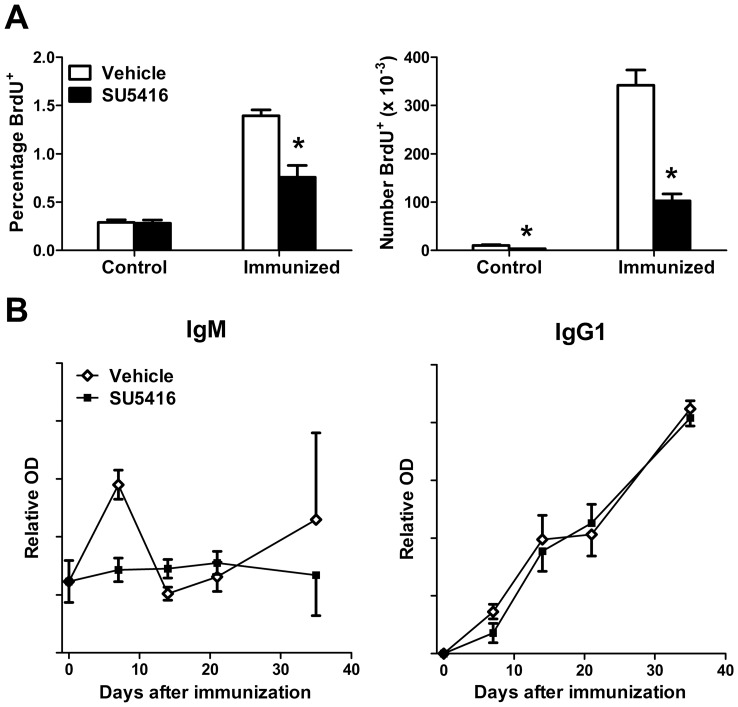
SU5416 treatment reduces immune responses. A) Mice were immunized unilaterally as described in Fig. 1. Mice were treated with SU5416 (25 mg/kg/day) or vehicle control starting on the day of immunization. After 3 days, mice were pulsed with BrdU for 1 hour, control and draining PLN were harvested and labeled with fluorochrome-conjugated antibodies to detect CD45 and BrdU, and analyzed by flow cytometry. Values represent the mean ± SEM percentage or number of CD45^+^ BrdU^+^ cells in each tissue from 4–5 mice per group. B) Mice were immunized in all limbs on days 0 and 28 with DNP-KLH-Alum and treated with SU5416 (50 mg/kg, twice per week) or vehicle control. Serum was collected 7, 14, 21 and 35 days following initial immunization and analyzed by isotype-specific ELISA for the presence of DNP-specific antibodies. Values represent the mean ± SEM levels of DNP-specific IgM and IgG1 antibodies from 5 mice per group. *Differences in the mean values between SU5416 and vehicle control treatments were significant; p<0.05.

The above results demonstrated that treatment with SU5416 led to significant corticosterone release. Since corticosterone is a well-known anti-inflammatory mediator, the effects of SU5416 treatment on cellular and humoral immune responses were examined. To determine the effects of SU5416 treatment on antigen-specific lymphocyte proliferation, mice were immunized with KLH-Alum, treated with SU5416 or vehicle control for three days and proliferation was measured with a 1 hour pulse of BrdU. As expected, both the frequency and number of BrdU^+^ cells were very low in the resting PLN, consistent with the low homeostatic proliferation rate of lymphocytes (Fig. 6A). While treatment with SU5416 significantly reduced the total number of BrdU^+^ cells in the resting PLN, it did not change their frequency. Thus, this finding likely resulted from the overall decrease in total cell numbers induced by SU5416 treatment (Fig. 1) and not from an actual reduction in basal lymphocyte proliferation. Immunization of PLN resulted in significant increases in the percentage and total number of BrdU^+^ cells in PLN (by 4.8- and 33-fold, respectively, Fig. 6A). However, within immunized PLN, SU5416 treatment significantly reduced both the frequency (by 46%) and number (by 70%) of BrdU^+^ cells (Fig. 6A). Therefore, SU5416 treatment dramatically reduced immunization-induced cellular proliferation in the draining PLN.

The effects of SU5416 treatment on the humoral immune response were also investigated. Specifically, mice were immunized with DNP-KLH-Alum, treated with SU5416 or vehicle control and boosted on day 28. Serum was collected on day 7, 14, 21 and 35 after the initial immunization and analyzed by ELISA for the presence of DNP-specific IgM and IgG1 antibodies. Results showed that treatment with SU5416 had only modest effects on the initial DNP-specific antibody response. Specifically, 7 days following immunization, vehicle-treated control mice showed a trend toward a greater DNP-specific IgM response than that of SU5416-treated mice (p = 0.12, Fig. 6B). In fact, there was no detectable DNP-specfic IgM response in SU5416-treated mice at any time point following immunization. Furthermore, SU5416-treated mice showed reduced levels of DNP-specific IgG1 (by 51%) 7 days following immunization although this did not reach statistical significance (p = 0.14). In addition, no significant differences were observed between groups of mice at later time points, including one week following a secondary immunization. Therefore, treatment with SU5416 had a much more profound effect on immunization-induced lymphocyte proliferation than on antibody production.

## Discussion

In this report, we have described off-target effects of a small-molecule RTK inhibitor, SU5416. Treatment with SU5416 increased serum corticosterone levels, negatively affected lymphocytes from primary and secondary lymphoid tissues, and reduced immune responses. In addition, we provide evidence that SU5416 may enhance corticosterone release directly from the adrenal glands by blocking the activation of TGF-β. These are previously undescribed attributes for SU5416 and need to be considered when using this compound.

SU5416 treatment significantly reduced PLN cellularity and induced the loss of progenitor lymphocytes in primary lymphoid tissues. Interestingly, during several clinical trials of SU5416, lymphopenia was reported as a grade 3 or 4 side effect in humans [15], [16], [17]. SU5416-induced glucocorticoid release could explain these effects. Specifically, glucocorticoids can induce apoptosis in double-positive thymocytes and bone marrow progenitor B cells [27], [32]. This effect likely explains the loss of thymocytes we observed following treatment with SU5416 (Fig. 3).

Injection of methylprednisolone has been reported to induce acute peripheral blood lymphopenia in humans and rodents, although this was thought to be the result of altered lymphocyte trafficking, and not peripheral cell loss [33], [34]. Indeed, normal blood cell numbers return approximately 24 hours after methylprednisolone injection, indicating that lymphocytolysis is most likely not accounting for the lymphopenia. In the current study, we observed reduced long-term accumulation of adoptively transferred lymphocytes into the PLN (Fig. 2B and C), which was not a result of detectable alterations in cell proliferation or apoptosis (data not shown). Since SU5416 was administered at the time of cell transfer during these long-term migration assays, the transferred cells were exposed to elevated levels of glucocorticoids in vivo. Therefore, glucocorticoid-induced alterations in lymphocyte trafficking may have sequestered the transferred cells in extravascular tissues (liver, lung, etc.). This phenomenon makes these cells unavailable for recirculation through and accumulation in PLN, and may explain the lack of lymphocyte accumulation in PLN in our studies [35].

The finding that B cells were the primary lymphocyte subtype in the periphery most affected by treatment with SU5416 was interesting given the severe effects of SU5416 treatment on thymocytes. However, the observed effect on B cells in the periphery may reflect differences in the respective turnover rates for T and B cells. Specifically, T cells typically have a much longer lifespan in the periphery than B cells [36]. Therefore, in the short term, a decreased output of new T cells would not be as apparent as a loss of replacement B cells; however, extended SU5416 treatment could result in a pronounced reduction in peripheral T cells. In addition to this possibility, glucocorticoids have been reported to induce a modest level of apoptosis of mature B cells [37]. Therefore, the reduction in peripheral B cells could be due to glucocorticoid-induced B cell apoptosis. Another explanation for the specific loss of peripheral B cells could be their glucocorticoid-induced sequestration in peripheral tissues such as liver or lung. As described above, it is known that glucocorticoids alter lymphocyte trafficking. B cells, in particular, may be sequestered in peripheral tissues to a greater extent or for a greater amount of time than other lymphocyte subsets.

Glucocorticoids are well-known anti-inflammatory mediators. Specifically, glucocorticoids reduce T cell activation through the dissociation of T cell receptor signaling complexes, and induce apoptosis of activated T cells [38], [39]. In addition, glucocorticoids can inhibit B cell activation [40]. Therefore, glucocorticoid release can account for the observed negative effects of SU5416 on immune responses in this study. Although SU5416 is a VEGFR inhibitor, the role of VEGF during immune responses remains controversial. Specifically, VEGF can have either pro- or anti-inflammatory properties, depending on the inflammatory context and target cell. For instance, VEGF can enhance T cell activation and differentiation into T helper type 1 (T_H_1) or T_H_17 effector cells, and can enhance inflammatory cytokine production [41], [42]. VEGF has also been reported to enhance inflammation in models of rheumatoid arthritis and psoriasis [43], [44]. Conversely, VEGF has been shown to induce endothelial cells to suppress T cell effector functions [45], and ectopic overexpression of VEGF in PLN significantly dampens humoral immune responses [24]. Therefore, the specific function of VEGF during immune responses remains unclear. Hence, the reduced immune responses observed following SU5416 treatment in the current study might be a result of both enhanced glucocorticoid levels and VEGFR blockade. However, since bevacizumab treatment had no effect on immunized tissues, specific blockade of VEGF/VEGFR likely has only a minor role in this process. It should also be noted that immunization alone may affect circulating corticosteroid levels [46], which could interact with the effects of SU5416 in ways that were not analyzed in this study. Regardless, the effects of SU5416 on acquired immunity in this report are consistent with acute low-dose administration of glucocorticoids. Specifically, acute glucocorticoid elevation has little to no effect on B cell antibody production [47], [48]. However, T cell proliferation in response to mitogen or antigen is significantly inhibited by acute low-dose glucocorticoids [49], [50], [51]. Therefore, the SU5416-induced immunosuppression observed in this report is consistent with acute low-dose glucocorticoid treatment.

While development of SU5416 as a cancer therapeutic is no longer ongoing, investigators continue to use SU5416 as a research tool. However, with continued investigation into VEGF-targeted treatments in many types of cancer, as well as macular degeneration, SU5416 may regain consideration as a therapeutic. Furthermore, several other small-molecule RTK inhibitors have been developed and are in use for both clinical and basic research [52]. Thus, it is important to reveal all potential effects of these molecules to better analyze and interpret the results of these studies.
